# Validation of NB CE-Chirps in the Diagnosis of Superior Semicircular Canal Dehiscence Syndrome

**DOI:** 10.3390/diagnostics16060868

**Published:** 2026-03-14

**Authors:** Quentin Mat, Christophe Lelubre, Antonino Maniaci, Stéphane Gargula, Giannicola Iannella, Jerome R. Lechien, Sophie Tainmont

**Affiliations:** 1 Department of Otorhinolaryngology, C.H.U. Charleroi, 6042 Charleroi, Belgium; 2 Department of Surgery, UMONS Research Institute for Health Sciences and Technology, University of Mons, 7000 Mons, Belgium; 3 Otology Study Group, Young Otolaryngologists-International Federation of Otorhinolaryngological Societies, 13005 Paris, France; 4 Department of Internal Medicine, C.H.U. Charleroi, 6042 Charleroi, Belgium; 5 Department of Medicine and Surgery, University of Enna Kore, 94100 Enna, Italy; 6 Department of Surgery, Asp 7 Ragusa, Modica Hospital, 97100 Ragusa, Italy; 7 Department of Otorhinolaryngology, La Conception University Hospital, Aix Marseille University, AP-HM, Centre National de la Recherche Scientifique (CNRS), Institut Universitaire des Systèmes Thermiques Industriels (IUSTI), 13005 Marseille, France; 8 Department of ‘Organi di Senso’, Sapienza University of Rome, 00042 Rome, Italy; 9 Department of Otolaryngology-Head Neck Surgery, Foch Hospital, Paris Saclay University, 92150 Paris, France

**Keywords:** superior semicircular canal dehiscence syndrome, vestibular evoked myogenic potential, narrow band CE-Chirp, air conduction, belly–tendon electrode montage

## Abstract

**Background/Objectives**: The aim of this study was to assess NB CE-Chirps for diagnosing Superior Semicircular Canal Dehiscence Syndrome (SSCDS) with cervical and ocular vestibular evoked myogenic potentials (cVEMPs and oVEMPs), and to compare them with Tone Bursts (TBs). **Methods**: Nine subjects diagnosed with SSCDS were included (four men/five women, median = 61 years, range = 31–79 years). Intensity thresholds at 500 Hz were investigated with both stimuli. A response was also sought when NB CE-Chirps and TBs were delivered at 4000 Hz for c and oVEMPs. **Results**: Both 500 Hz TBs and 500 Hz NB CE-Chirps significantly differentiated affected ears from healthy ears for cVEMPs (*p* < 10^−3^ in both cases) and oVEMPs (*p* < 10^−3^ in both cases). Furthermore, we observed significantly lower intensity thresholds in SSCDS ears with 500 Hz NB CE-Chirps than with 500 Hz TBs for both cVEMPs (*p* < 10^−3^) and oVEMPs (*p* = 0.036). Regarding the response rate at 4000 Hz, only TBs consistently showed a response in 100% of cases for the affected ears, with no response in healthy ears for both cVEMPs and oVEMPs. However, there was no significant difference between the response rates obtained at 4000 Hz using TBs and NB CE-Chirps in affected ears (*p* = 1.000 for cVEMPs and *p* = 1.000 for oVEMPs). **Conclusions**: Searching for intensity thresholds with NB CE-Chirps 500 Hz in cVEMPs and oVEMPs is an effective method for diagnosing SSCDS, likely with better frequency specificity than with 500 Hz TBs. Stimulation at 4000 Hz with both TBs and NB CE-Chirps appears to be a promising test for easily screening this syndrome, reducing both sound exposure and the duration of the examination. The possibility to reduce rise time in 4000 Hz TBs may favor this stimulus over NB CE-Chirps at this frequency for this disease. These results should be confirmed in larger cohorts including patients with more severe forms.

## 1. Introduction

Superior Semicircular Canal Dehiscence Syndrome (SSCDS) is a rare condition first described by Lloyd B. Minor and colleagues in 1998 [1]. It is characterized by vestibular and cochlear phenomena related to the presence of a bony defect in the roof of the superior semicircular canal [2,3]. The diagnosis is primarily based on the detection of lowered intensity thresholds in vestibular evoked myogenic potentials (VEMPs) and confirmation of the dehiscence through high-resolution CT scan or cone beam imaging of the petrous bone [3,4,5,6]. Additionally, recent data have suggested that patients with SSCDS can exhibit a VEMP response at 4000 Hz in nearly 100% of cases on the affected side, while no response is recorded in the healthy ear at this frequency [4,7]. There are two types of VEMPs: ocular vestibular evoked myogenic potentials (oVEMPs) and cervical vestibular evoked myogenic potentials (cVEMPs). cVEMPs were first described by Colebatch et al. in 1994. They assess saccular function and the ipsilateral sacculo-colic pathway [8]. They correspond to a relaxation of the ipsilateral sternocleidomastoid (SCM) muscle to the stimulated saccule [6,8]. oVEMPs assess utricular function and the controlateral vestibuloocular pathway [6,9]. They correspond to the measurement of a contraction in the contralateral inferior oblique oculomotor muscle to the stimulated utricle [6,9]. The most commonly used stimulus for eliciting VEMPs is the Tone Burst (TB) [6]. However, recent studies have shown that Narrow Band Claus Elberling-Chirps (NB CE-Chirps) produce larger amplitudes during oVEMPs at a frequency of 500 Hz with an intensity of 100 dB normalized hearing level (nHL) and during cVEMPs at 500 Hz with an intensity of 95 dB nHL, compared to TBs in groups of healthy adult subjects [10,11,12]. NB CE-Chirps have also reduced the incidence of false-negative responses in cVEMPs [12]. Therefore, it would be valuable to validate NB CE-Chirps in peripheral vestibular disorders to potentially allow for its use in routine clinical practice to be recommended. The aim of this study was to prospectively compare NB CE-Chirps 500 and 4000 Hz to TBs for the diagnosis of SSCDS with c and oVEMPs. To our knowledge, this is the first study investigating NB CE-Chirps in SSCDS using c and oVEMPs.

## 2. Materials and Methods

### 2.1. Participants

Nine subjects with SSCDS were included in this prospective study (4 men/5 women, median = 61 years, range = 31–79 years). One patient presented with a bilateral form of SSCDS, resulting in a total of 10 affected ears and 8 healthy ears.

All these patients had previously been diagnosed based on clinical history and a cone beam CT of the temporal bones, along with an assessment of the intensity thresholds of cVEMPs and oVEMPs using 500 Hz TBs. They were then invited to participate in this study.

Since some elements may alter the recording of air-conducted VEMPs, exclusion criteria were defined (Table 1).

Patients underwent a standardized audiological assessment to determine eligibility for the study. This evaluation included micro-otoscopy (Zeiss OPMI Pico, Zeiss, Oberkochen, Germany) and tympanometry with acoustic reflex measurements (GSI Tympstar Grason-Stadler, Eden Prairie, MN, USA). Hearing thresholds were assessed by pure-tone audiometry for both air and bone conduction using TDH-39 headphones and a B71 bone vibrator (Equinox, Interacoustics, Middelfart, Denmark). Speech audiometry was carried out using the French Fournier disyllabic word lists (Equinox, Interacoustics, Middelfart, Denmark). The last two examinations were performed in a sound-treated booth (Boët Stopson, Villeneuve d’Ascq, France).

For each participant, air conduction (AC) and bone conduction (BC) pure-tone averages (PTAs) were calculated for both the affected and contralateral ears. The PTA was defined as the mean hearing threshold measured at 500, 1000, 2000, and 4000 Hz during pure-tone audiometry.

Participants were also asked to complete a prevalence questionnaire to identify the most frequently experienced symptoms. The list of symptoms investigated is provided in Table 2.

### 2.2. Recording Procedure

To minimize sound exposure, the oVEMPs and cVEMPs were carried out on two days separated by a minimum of 48 h.

#### 2.2.1. oVEMPs

All recordings were performed in a sound-attenuated and Faraday-shielded booth (BERA, Boët StopSon, Villeneuve d’Ascq, France). Participants sat on a height-adjustable chair, allowing the examiner to standardize head and gaze position across individuals. During testing, subjects were instructed to relax their jaw and to fix their gaze on a bright red target positioned on the opposite wall at a distance of 2 m. This fixation point was placed so that the visual axis formed an angle of 30° above the horizontal plane. Before electrode placement, the skin was cleaned and gently abraded using Nuprep Skin Prep Gel (Weaver and Company, Aurora, CO, USA) and Ether^®^. Disposable surface electrodes (133 Foam Electrodes, Covidien™, Mansfield, MA, USA) were then applied. Recordings were obtained using a contralateral belly–tendon electrode montage (BTEM). The active electrode was positioned about 1 cm below the lower eyelid margin contralateral to acoustic stimulation and slightly lateral to the center of the eye, corresponding to the cutaneous projection of the inferior oblique muscle. The reference electrode was placed at the medial canthus, while the ground electrode was positioned on the forehead [13,14]. Electrode impedance was maintained below 5 kΩ, with interelectrode impedance kept below 3 kΩ. Acoustic stimulation was delivered through insert earphones (INSERT 3M E-A-RTONE™ 3A, Minneapolis, MN, USA). Stimulus presentation and recording of the evoked myogenic responses were performed using the Eclipse EP25 system (Interacoustics, Assens, Denmark), calibrated according to the International Organization for Standardization standard ISO 389-6.

Each ear was tested by TBs (2-2-2 ms) and NB CE-Chirps (9 ms) at 500 Hz with a starting intensity of 95 dB nHL and decrement steps of 5 dB until reaching the thresholds. The threshold was defined as the smallest sound intensity allowing for oVEMP recording. The response was considered present if the recorded curve was greater than the signal noise. The n1 and p1 waves were identified at the latencies usually described for these stimuli [6,11,15,16]. One hundred stimulations were delivered for each recording.

Acoustic stimuli were presented at a repetition rate of 5.1/s using rarefaction polarity and a band-pass filter of 1–1000 Hz [6,17,18]. Electrical activity was recorded within a time window extending from 20 ms prior to stimulus onset to 80 ms after stimulation. To ensure the reliability of the recordings, each measurement was performed twice and the resulting traces were averaged. A one-minute rest period was provided to participants between successive recordings. Thereafter, the same sounds were delivered at 4000 Hz (TB: 0-2-0 ms and NB CE-Chirp: 2.5 ms) with an intensity of 95 dB nHL. The running order was randomized (right or left ear first; NB CE-Chirps or TB first).

#### 2.2.2. cVEMPs

cVEMPs were performed in the same cabin with the same equipment and the same sound stimulations. Participants were also seated upright. Here are reported some features related to cVEMPs.

For cVEMP recordings, the active electrode was positioned over the middle third of the sternocleidomastoid (SCM) muscle on the side of acoustic stimulation. The reference electrode was placed on the manubrium, while the ground electrode was located at the midline of the forehead. Each ear was evaluated separately using air-conducted stimuli in order to obtain ipsilateral myogenic responses. Prior to stimulus delivery, participants were instructed to rotate their head toward the side opposite to the stimulated ear and to maintain this position throughout the recording period. To ensure constant muscle activation, participants received real-time visual feedback of ipsilateral SCM contraction via a monitoring screen displaying electromyographic (EMG) activity. This feedback allowed for comparable levels of contraction to be achieved for both sides. Electromyographic activity was monitored using the same surface electrodes employed for cVEMP recording, and acceptable contraction levels ranged between 50 and 150 μV.

Moreover, amplitude normalization was also carried out before identifying the curves, so that the amplitude of the analyzed curves was no longer dependent on a potential asymmetry of contraction of the SCM muscles (corrected amplitudes). This part is essential for obtaining reliable cVEMPs.

Acoustic stimuli were delivered at a rate of 5.1 stimuli per second using rarefaction polarity, and the recorded signals were processed with a band-pass filter ranging from 10 to 750 Hz [6,18,19]. The number of stimulations was 200 times per acquisition.

### 2.3. Statistical Analysis

The responses from the participants to the questionnaire on the prevalence of the various symptoms of SSCDS are represented as percentages in the form of bar charts. Normality was assessed using QQ plots and the Shapiro–Wilk test for inferential statistics. Therefore, a paired samples T-test was used to compare AC and BC PTA obtained in healthy and pathological ears. Comparisons of intensity thresholds obtained in c and oVEMPs with 500 Hz TB and NB CE-Chirps were performed using Friedman’s test with the Dunn–Bonferroni procedure for post hoc multiple comparisons. Finally, the response rates obtained in c and oVEMPs with TBs and NB CE-Chirps at the frequency of 4000 Hz were compared using Cochran’s Q test and Bonferroni correction for multiple comparisons. Only unilateral forms of SSCDS were included for inferential statistics. All tests were two-sided with an alpha error level of 0.05. A *p* < 0.05 was considered significant. Statistical analyses were performed using JASP Team (2024) software (JASP (Version 0.18.3)) and IBM^®^ SPSS^®^ Statistics version 23.0 (IBM, Ehningen, Germany).

## 3. Results

### 3.1. Prevalence of Key Symptoms Associated with SSCDS

**Figure 1:**
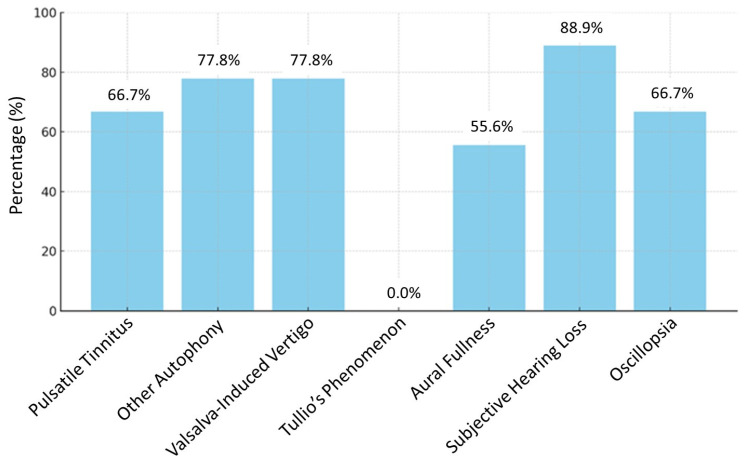
Bar charts of SSCDS-related symptoms among the 9 patients who responded to the prevalence questionnaire. Results are expressed as the percentage of patients reporting these symptoms.

The prevalence of the various symptoms investigated in our patient cohort is presented in Figure 1.

### 3.2. Comparison of PTAs Obtained in Air and Bone Conduction in Healthy and Pathological Ears

**Figure 2:**
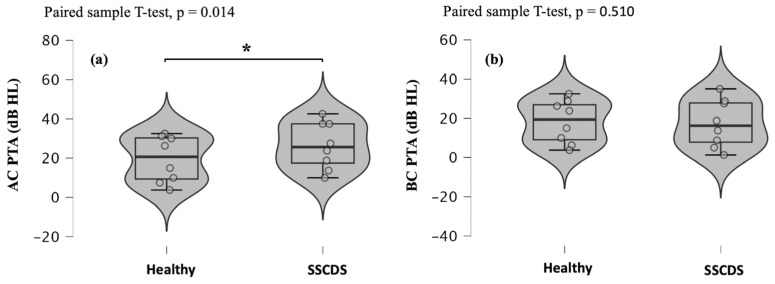
Violin plots and box plots showing air-conducted (**a**) and bone-conducted (**b**) pure-tone averages obtained in ears with SSCDS and healthy ears. The pure-tone average was calculated from the average of the air-conducted (**a**) and bone-conducted (**b**) hearing thresholds obtained from 500, 1000, 2000, and 4000 Hz during pure-tone audiometry.

AC PTAs were significantly higher in the affected ears compared to the healthy ears (*p* = 0.014; paired sample T-test) (Figure 2a). In contrast, no significant difference was observed in the BC PTAs between the healthy ears and those with SSCDS (*p* = 0.510; paired sample T-test) (Figure 2b).

### 3.3. Comparison of Intensity Thresholds Obtained in Healthy and Pathological Ears Using 500 Hz NB CE-Chirp and 500 Hz TB Stimuli in cVEMPs

**Figure 3:**
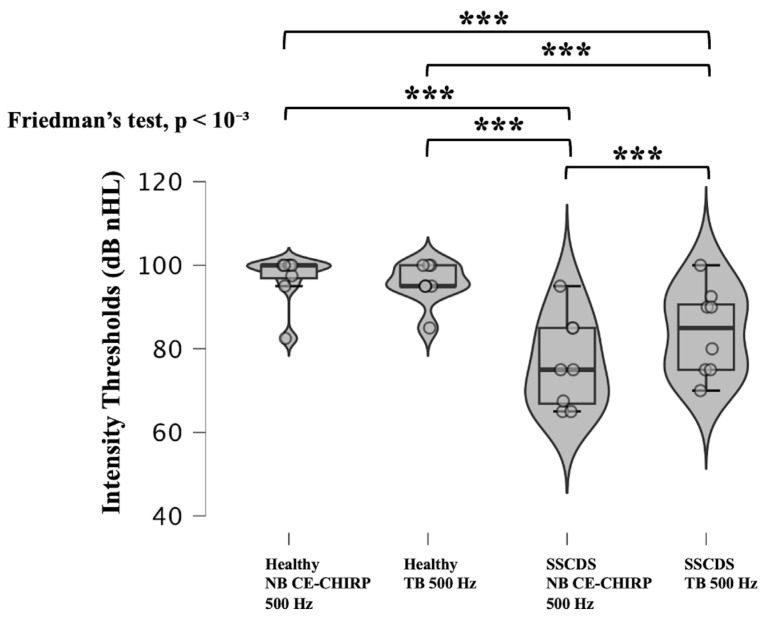
Violin plots and box plots showing intensity thresholds obtained with cVEMPs for healthy and SSCDS ears with 500 NB CE-Chirp and 500 Hz TB. All pairwise comparisons are topped with * when they are significantly different (Dunn–Bonferroni procedure).

Significant differences in intensity thresholds were observed depending on the ear tested and the type of auditory stimulus used (*p* < 10^−3^; Friedman’s test) (Figure 3). Both 500 Hz NB CE-Chirps and 500 Hz TB stimuli elicited significantly lower cVEMP thresholds in pathological ears compared to healthy ears (*p* < 10^−3^ for all comparisons; Dunn–Bonferroni post hoc test). While no significant difference was found between thresholds obtained with NB-CE-Chirp and TB stimuli in healthy ears (*p* = 1.000; Dunn–Bonferroni), pathological ears showed significantly lower thresholds with the 500 Hz NB CE-Chirp compared to the 500 Hz TB (*p* < 10^−3^; Dunn–Bonferroni).

### 3.4. Comparison of Intensity Thresholds Obtained in Healthy and Pathological Ears Using 500 Hz NB CE-Chirp and 500 Hz TB Stimuli in oVEMPs

**Figure 4:**
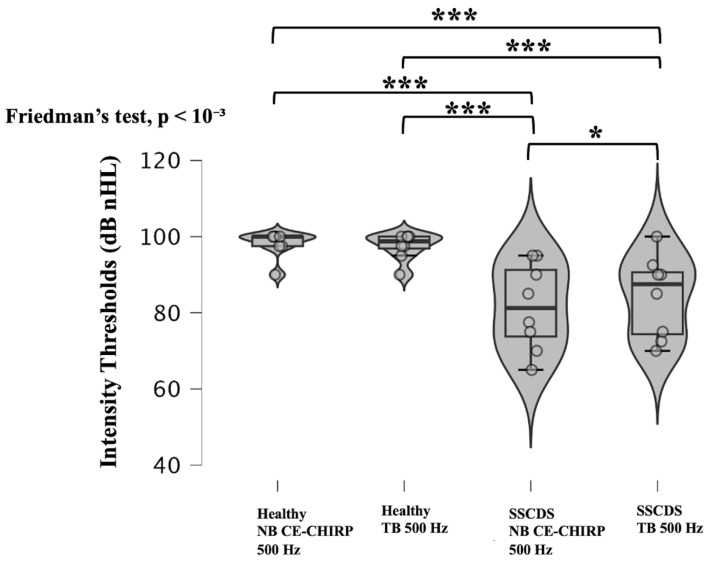
Violin plots and box plots showing intensity thresholds obtained with oVEMPs for healthy and SSCDS ears with 500 NB CE-Chirps and 500 Hz TBs. All pairwise comparisons are topped with * when they are significantly different (Dunn–Bonferroni procedure).

Once again, significant differences were observed between the ears tested and stimuli used (*p* < 10^−3^; Friedman’s test) (Figure 4). Both the 500 Hz NB CE-Chirp and 500 Hz TB stimuli were able to differentiate healthy ears from ears with SSCDS, which exhibited significantly lower cVEMP thresholds (*p* < 10^−3^ for all comparisons; Dunn–Bonferroni post hoc test). The 500 Hz NB CE-Chirps also produced significantly lower intensity thresholds than the 500 Hz TBs in pathological ears (*p* = 0.036; Dunn–Bonferroni). No significant difference was observed between thresholds obtained with NB CE-Chirps and TBs in healthy ears (*p* = 1.000; Dunn–Bonferroni).

### 3.5. Response Rates at 4000 Hz Using TBs and NB CE-Chirps for cVEMPs

Indeed, we observed significantly higher response rates on the pathological side with both 4000 Hz TB and NB CE-Chirp stimuli (*p* < 10^−3^; Cochran’s Q test) (*p* = 0.003 for TBs and NB CE-Chirps; Dunn–Bonferroni post hoc test). No healthy ears showed a response at 4000 Hz with either TBs or NB CE-Chirps, whereas responses at 4000 Hz were present in 100% of cases on the pathological side with both TBs and NB-CE-Chirps (Table 3).

### 3.6. Evaluation of Response Rates at 4000 Hz Using TB and NB CE-Chirp Stimuli for oVEMPs

Once again, significant differences were observed in the 4000 Hz oVEMP response rates depending on the ear tested (*p* < 10^−3^; Cochran’s Q test). No healthy ears responded at 4000 Hz with either TB or NB CE-Chirp stimuli. Among the pathological ears, eight out of eight showed a response at 4000 Hz with TBs, and seven out of eight responded with NB CE-Chirps. However, there was no significant difference in response rates between TBs and NB CE-Chirps at 4000 Hz in pathological ears (*p* = 1.000; Dunn–Bonferroni test) (Table 4).

## 4. Discussion

SSCDS is a rare condition caused by the absence of bony coverage of the superior semicircular canal [2,3]. Patients may complain of brief dizziness triggered by valsalva or loud sounds (Tullio’s phenomenon) or chronic disequilibrium [1,3,20]. Hearing loss and oscillopsia may also be reported [3]. They sometimes describe autophony like pulsatile tinnitus perceived in the affected ear [2,3]. Hearing loudly one’s eye movements or blinking, borborygmi, neck movements, and footfalls are other types of autophony [3]. In our patient series, we found that all of these symptoms were frequently reported and subjective hearing loss was the most common. Interestingly, none of the patients reported Tullio’s phenomenon. A recent study evaluating the presence of symptoms related to this syndrome showed that vestibular symptoms were more pronounced in bilateral forms [21]. The absence of Tullio’s phenomenon reported in this study could be because mostly unilateral forms were included. Furthermore, the patients selected for this study did not require surgical treatment. It is possible that Tullio’s phenomenon was not reported in this cohort because it is usually disabling and requires surgical management.

Regarding hearing loss, a low-frequency air-bone gap can be identified with pure-tone audiometry [2,3,20,22]. This could be caused by an improvement in bone conduction but also elevated air-conducted hearing thresholds due to a partial dissipation of acoustic energy through dehiscence [22]. In this study, we indeed observed a significant elevation of air-conducted hearing thresholds on pure-tone audiometry on the affected side. However, we did not record decreased bone-conducted hearing thresholds in the pathological ears. The lack of statistical significance may be explained by the small sample size.

Moreover, performing c and oVEMPs is essential for establishing the diagnosis of SSCDS [3]. Indeed, SSCDS is associated with increased amplitudes in the affected ear at classical stimulation intensities in c and oVEMPs at the frequency of 500 Hz [4,5,6]. Because of these larger amplitudes, the intensity thresholds for the appearance of c and oVEMPs are lowered [4,5,6]. The underlying pathophysiological mechanism could be that dehiscence could improve the amount of sound energy reaching the vestibule, which would increase the activation of the otolith organs but would also allow the activation of the primary irregular semicircular canal afferent neurons [4]. The search for a response at 4000 Hz has recently been introduced and appears to be advantageous for its speed of execution and its greater ease for detecting bilateral forms [4,7].

Currently, the 500 Hz TB is the most widely used stimulus for VEMPs. Indeed, these sounds have demonstrated better amplitudes as well as better response rates in the healthy population due to their better frequency selectivity and their longer duration compared to clicks [18,19]. Recent research has shown that NB CE-Chirps produce larger amplitudes for oVEMPs at 500 Hz with an intensity of 100 dB nHL and for cVEMPs at 500 Hz with an intensity of 95 dB nHL compared to TBs in groups of healthy adult subjects [10,11,12]. They have also been found to reduce the rate of false-negative responses in cVEMPs [12]. These superior results could be explained by the absence of spectral splatter with NB CE-Chirps at 500 Hz unlike TBs at 500 Hz and thus a better frequency selectivity [12,23]. Therefore, validating this stimulus across various peripheral vestibular disorders, including SSCDS, would support its adoption in routine clinical practice.

The primary aim of this study was to clinically validate NB CE-Chirps in the diagnosis of SSCDS. A secondary objective was to explore ways to optimize the diagnosis of this syndrome to facilitate its detection.

To this end, we compared intensity thresholds obtained using 500 Hz TBs for cVEMPs and oVEMPs with those obtained using 500 Hz NB CE-Chirps. Our findings indicate that the 500 Hz NB CE-Chirp, like the 500 Hz TB, can effectively differentiate a healthy ear from one affected by SSCDS. Furthermore, we observed significantly lower intensity thresholds with 500 Hz NB CE-Chirps than with 500 Hz TBs on the pathological side. These lower thresholds could be explained by the better frequency specificity of the 500 Hz NB CE-Chirps [11,12,23]. Therefore, 500 Hz NB CE-Chirps appear to be effective stimuli, as they allow for the identification of SSCDS with intensity thresholds even lower than with 500 Hz TBs in c and oVEMPs. These lower thresholds could allow for a decrease in the starting sound intensity when searching for SSCDS, reducing the risk of cochlear damage.

Moreover, we did not observe any responses at 4000 Hz in healthy ears using either the TB or the NB CE-Chirp for c and oVEMPs. Regarding the pathological ears, 100% of the affected ears showed a 4000 Hz response in TB for c and oVEMPs and one affected ear was not identified using the 4000 Hz NB CE-Chirp for oVEMPs. Although there was no significant difference observed in this small sample, using a 4000 Hz TB could have some advantages compared to the 4000 Hz NB CE-Chirp to identify SSCDS. Indeed, while the resonance frequency of the middle ear–vestibular system is around 500 Hz, the dehiscence of the superior semicircular canal allows otolith and superior canal stimulation at higher frequencies such as 4000 Hz by diverting sound energy to the vestibule [4,7,18,19,24,25]. However, even under these conditions, 4000 Hz remains a suboptimal stimulation frequency for otolith organs and the superior semicircular canal compared to 500 Hz; this explains the lower amplitudes of the responses recorded with NB CE-Chirps and TBs at 4000 Hz in comparison with those obtained at 500 Hz in c and oVEMPs [4,26]. Therefore, the rise time could be the main factor allowing the recording of c and oVEMPs at 4000 Hz rather than the frequency specificity of the delivered stimulus [4,7]. Indeed, it is the type 1 hair cells of the striolar region of the macula that generate the c and oVEMPs [27,28,29,30]. These cells are in contact with afferent neurons with irregular resting discharge [28,31]. These type I hair cells, like those in the ampullary crests of semicircular canals, are sensitive to large accelerations, such as impulses [27,28,29,30]. Short rise times can thus be considered strong impulses because they allow for the delivery of maximum sound intensity as quickly as possible, thereby causing a greater displacement of the ciliary bundle of type I cells of the striolar part of macula [4,7]. Since TBs have the advantage of being defined by their time domain, the rise times of 0 ms (0-2-0 ms) with the 4000 Hz TBs could explain why 100% of pathological ears were detected using cVEMPs and oVEMPs, whereas the 4000 Hz NB CE-Chirps missed one pathological ear in oVEMPs due to their longer rise time (1.5 ms). Moreover, it has also been shown that shorter rise times increase the recorded amplitudes at 500 Hz using TBs for oVEMPs in healthy populations, which reinforces this theory [32]. This hypothesis, however, needs to be confirmed by larger series of SSCDS including more severe and bilateral forms.

In addition, the use of a 4000 Hz stimulus does not require searching for intensity thresholds, which reduces the duration of the examination and the sound energy delivered, making the test less disturbing for certain patients who sometimes present with hyperacusis or Tullio’s phenomenon. Therefore, the choice of acoustic stimulus in c and oVEMPs should be tailored to the selected detection approach for SSCDS. Threshold-based protocols are likely to be more effective using 500 Hz NB CE-chirps, whereas detection testing at 4000 Hz would be more appropriately performed with TBs due to their impulse nature.

Finally, as reported by Makowiec and colleagues, using a BTEM for oVEMPs proved effective in differentiating a healthy ear from a pathological ear with SSCDS [33]. Further analyses on larger cohorts are necessary to confirm these findings.

## 5. Conclusions

Intensity threshold testing with 500 Hz NB CE-Chirps during c and oVEMPs is an effective method for diagnosing SSCDS. Stimulation at a frequency of 4000 Hz with TB and NB CE-Chirps appears to be a promising test for easily detecting this syndrome by reducing sound exposure and examination duration. The possibility of reducing rise time with 4000 Hz TB could be an element favoring this stimulus over NB CE-Chirp 4000 Hz. These results need to be confirmed in larger groups including severe presentations and bilateral cases.

## Figures and Tables

**Table 1:** Exclusion criteria.

Exclusion Criteria
Not having previously been operated on for SSCDS
Additional anomaly to superior semicircular canal dehiscence on a cone beam CT scan of the petrous bone (e.g., dehiscence of another semicircular canal, large vestibular aqueduct, otosclerosis, other ossicular ankylosis, ossicular dislocation or fracture, incomplete ossicular chain, middle ear filling, middle ear mass, major or minor ear aplasia, external auditory canal mass or infection, tympanic membrane perforation, …).
Neurological or muscular pathology impairing myogenic responses
Previous oculomotor problems
Previous otologic surgery
Taking ototoxic or myorelaxant medication
Type 1 or 2 diabetes mellitus

**Table 2:** Prevalence questionnaire.

Prevalence Questionnaire
Pulsatile tinnitus, and the affected side
Any other form of autophony (e.g., eye movement, footstep sounds, borborygms)
Vertigo during Valsalva maneuver
Vertigo in response to loud sounds (Tullio’s phenomenon)
Ear fullness
Oscillopsia
Subjective hearing loss

**Table 3:** Response rates in pathological and healthy ears with TBs and NB CE-Chirps at 4000 Hz for cVEMPs.

Stimulus Type	Pathological Ears (SSCDS)	Healthy Ears
TB 4000 Hz	100% (8/8)	0% (0/8)
NB CE-Chirp 4000 Hz	100% (8/8)	0% (0/8)

**Table 4:** Response rates in pathological and healthy ears with TBs and NB CE-Chirps at 4000 Hz for oVEMPs.

Stimulus Type	Pathological Ears (SSCDS)	Healthy Ears
TB 4000 Hz	100% (8/8)	0% (0/8)
NB CE-Chirp 4000 Hz	87.5% (7/8)	0% (0/8)

## Data Availability

The datasets generated and/or analyzed during the current study are available from the corresponding author upon reasonable request.

## Abbreviations

The following abbreviations are used in this manuscript:

NB CE-Chirp	Narrow Band Claus Elberling-Chirp
SSCDS	Superior Semicircular Canal Dehiscence Syndrome
VEMP	Vestibular evoked myogenic potential
cVEMP	Cervical vestibular evoked myogenic potential
oVEMP	Ocular vestibular evoked myogenic potential
TB	Tone Burst
Hz	Hertz
SCM	Sternocleidomastoid
dB HL	Decibel hearing level
nHL	Normalized hearing level
AC	Air-conducted
BC	Bone-conducted
PTA	Pure-tone average
BTEM	Belly–tendon electrode montage
EMG	Electromyography
